# A Review of Modern Imaging Landscape for Prostate Cancer: A Comprehensive Clinical Guide

**DOI:** 10.3390/jcm12031186

**Published:** 2023-02-02

**Authors:** Paul Gravestock, Bhaskar Kumar Somani, Theodoros Tokas, Bhavan Prasad Rai

**Affiliations:** 1 Department of Urology, Freeman Hospital, Newcastle upon Tyne NE7 7DN, UK; 2 Department of Urology, University Hospital Southampton NHS Trust, Southampton SO16 6YD, UK; 3 Department of Urology and Andrology, General Hospital Hall in Tirol, 6060 Hall in Tirol, Austria; 4 Training and Research in Urological Surgery and Technology (T.R.U.S.T.)-Group, 6060 Hall in Tirol, Austria

**Keywords:** prostatic neoplasms, image-guided biopsy, multiparametric magnetic resonance imaging, positron-emission tomography

## Abstract

The development of prostate cancer imaging is rapidly evolving, with many changes to the way patients are diagnosed, staged, and monitored for recurrence following treatment. New developments, including the potential role of imaging in screening and the combined diagnostic and therapeutic applications in the field of theranostics, are underway. In this paper, we aim to outline the current landscape in prostate cancer imaging and look to the future at the potential modalities and applications to come.

## 1. Introduction

Imaging for prostate cancer has developed significantly over the last two decades. There has been a range of modalities utilized, including the recent application of functional imaging. Additionally, indications for imaging have expanded beyond diagnostics to biopsy guidance, staging and risk stratification, active surveillance, and the detection of recurrence. Furthermore, there are developing roles for imaging in population screening and theranostic applications. We aim to provide an up-to-date narrative looking at the role of prostate cancer imaging in these key areas.

## 2. Imaging in Prostate Cancer Diagnostics

Historically, prostate cancer has been difficult to detect and biopsy. Digital rectal examination (DRE) was the mainstay of diagnosis, with transrectal biopsies targeting palpable lesions [1]. This blind approach was superseded by the development of transrectal ultrasound (TRUS). TRUS utilizes a rectal ultrasound probe to directly visualize the prostate. However, whilst prostate cancer can appear hypoechoic on an ultrasound, it is not an accurate detection method, and as a diagnostic or staging modality, it performs poorly [2,3]. As a result, systematic prostate biopsies in patients with suspected prostate cancer using TRUS to guide the needle became the diagnostic standard. These were initially performed using a sextant biopsy protocol that involved taking three biopsy cores from each side of the prostate. Due to high false negative rates, this was refined to what is termed an extended protocol involving 10–12 total cores, which was found to have an increased sensitivity for prostate cancer detection [4]. However, systematic biopsies that are performed based on DRE findings or prostate specific antigen (PSA) levels do not discriminate well between clinically significant and clinically insignificant cancers. One tool that has been subsequently introduced to improve diagnostic accuracy is multiparametric MRI (mpMRI).

mpMRI has been shown to be highly sensitive in the diagnosis of prostate cancer. The PROMIS trial found mpMRI to have a sensitivity of 93%, estimating that its introduction into the diagnostic pathway would avoid up to 27% of patients undergoing an initial prostate biopsy and result in fewer clinically insignificant cancer diagnoses [5]. It has subsequently become the first line imaging used in the diagnosis of localized prostate cancer, with patients found to have a raised PSA level or abnormal DRE routinely undergoing mpMRI prior to prostate biopsy [6]. mpMRI consists of a combination of MRI sequences; anatomical sequences providing detail of the prostate, in particular T2-weighted (T2W) images, and functional sequences including diffusion weighted imaging (DWI) and dynamic contrast enhancement (DCE), to assess for features suspicious for potential prostate cancer [7]. The interpretation is standardized, using either the Prostate Imaging-Reporting and Data System (PI-RADS) or Likert scoring systems that aim to quantify the likelihood of clinically significant prostate cancer being present on a scale of 1 to 5 [7,8].

There is ongoing debate in the relation to mpMRI for prostate cancer diagnostics regarding the use of DCE, and to whether T2W and DWI alone, termed biparametric MRI (bpMRI), is sufficient. bpMRI has been shown to be non-inferior to mpMRI and has the advantage of being less time-consuming while removing the risk associated with contrast media, though at present there remains no clear consensus [9,10,11,12].

### 2.1. Screening

Prostate cancer screening itself is a contentious issue. Screening using PSA has been examined by several large trials. The CAP trial in the United Kingdom (UK) examined prostate cancer screening in over 400,000 men who underwent a single PSA test ± biopsy when raised and found no significant difference in prostate cancer mortality between those who underwent PSA screening and the control arm at 10 years [13]. The European Randomized Study of Screening for Prostate Cancer (ERSPC) followed up with over 180,000 men undergoing regular PSA screening (between two and seven yearly intervals) with biopsies if raised and found that prostate cancer mortality was reduced in the screened arm. However, this was associated with a high number needed to diagnose of 1 in 48 to avert one prostate cancer death at a follow-up of 9 years, though this was reduced to 1 in 18 at 16 years showing an increased benefit with a longer follow-up [14]. Despite this improvement in mortality that is seen with PSA screening, it comes at the cost of considerable overdiagnosis, and potential overtreatment, of clinically insignificant prostate cancer.

As discussed previously, mpMRI has been shown to reduce the number of clinically insignificant cancers diagnosed, and on this basis, screening using an MRI has been investigated. In the IP1-PROSTAGRAM trial, 408 men underwent a PSA, MRI, and TRUS (B-mode and shear wave elastography); men who were deemed to be positive in any of the three tests (reporters of imaging were blinded to PSA) underwent a systematic transperineal (TP) biopsy + targeted cores if they were found to be positive at MRI or TRUS. In this trial, MRI was found to detect more clinically significant and less clinically insignificant cancers than PSA alone [15]. Other trials are in progress further examining this. ReIMAGINE is a study assessing screening for prostate cancer within a UK population, randomly inviting around 300 eligible men aged 50–75 for an MRI to assess the feasibility of screening and prevalence of MRI-detected suspicious lesions in the general population, for which recruitment was completed in December 2020 [16]. Additionally, MRI vs. PSA (MVP) is a Canadian randomized controlled trial awaiting publication that compares men undergoing screening via PSA with a subsequent biopsy if raised vs. those screened with an MRI and followed up by US-MRI fusion biopsy if abnormal lesions are detected [17]. Of note, all three of the above trials used bpMRI.

### 2.2. Biopsies

Prostate biopsies are used to further assess patients with suspicious mpMRI results in the context of other factors, such as PSA and DRE findings. Biopsies were predominately undertaken by a transrectal (TR) approach, though more recently, there has been a shift toward TP biopsies. This has been driven by an increasing body of evidence showing lower infectious complications and a reduced antibiotic prophylaxis requirement for TP biopsies, with some studies suggesting that, in select patients, no antibiotic prophylaxis is required at all [18,19,20,21,22]. As a result, this is reflected in international guidance with the European Association of Urology (EAU) guidance, which strongly recommends using a TP approach [6]. Additionally, TRANSLATE is a randomized control trial (RCT) looking to definitively address which approach is better, directly comparing local anaesthetic (LA) TP prostate biopsy with LA TR prostate biopsy. The primary outcome evaluated will be the detection of clinically significant prostate cancer, with secondary outcomes including infection rates, tolerability, complications, cost effectiveness, and the need for repeated biopsies [23].

Prostate biopsies can be used to target suspicious MRI lesions or systematically sample the prostate. The PRECISION trial showed that when men with a clinical suspicion for prostate cancer underwent mpMRI followed by targeted biopsies, there was a higher detection rate for clinically significant prostate cancer and less clinically insignificant prostate cancers detected than those who underwent indiscriminate systematic biopsies [24]. Further studies, including 4M, MRI-FIRST, and PAIREDCAP, have shown that the best detection rates are achieved by combining systematic and targeted biopsies with the omission of either set shown to miss a proportion of clinically significant cancers. For example, in patients undergoing MR-targeted biopsies, the addition of systematic biopsies yielded the detection of 7% extra clinically significant cancers in the 4M trial and 5.2% in MRI-First. As a result, a combined approach is recommended [6,25,26,27]. Key trials examining mpMRI- and MR-targeted biopsies (TB) that were compared with systematic biopsies (SB) are summarized in Table 1.

Transrectal ultrasound allow for the visualization of the prostate anatomy and the biopsy needle, usually in both the axial and sagittal planes. Targeted biopsies can be performed using a cognitive approach whereby the operator reviews the MRI imaging and estimates the corresponding area on TRUS imaging. Alternatively, fusion software can be used to directly superimpose the suspicious areas seen on the MRI over the TRUS images. Novel robotic solutions have been developed in conjunction with fusion software to increase targeting accuracy. These include systems that use a robotic needle guide to target the suspicious lesion, defining the position and depth with the operating surgeon only required to insert the biopsy gun and fire [28].

The limitations of fusion-guided biopsies are primarily related to the process of accurately overlaying the MRI targets onto the live TRUS images, thereby methods have been developed to cut out the TRUS middleman in the form of in-bore MRI biopsies [29]. An in-bore MRI biopsy involves a rectal needle guide and sequential MRI imaging with the patient removed from the scanner and the needle guide adjusted, with further MRI sequences performed until it is adequately aligned with the area of interest. At this point, the patient can be removed from the scanner and a biopsy can be taken, with the option for confirmatory re-imaging if required [30]. Again, novel robotic solutions have been developed to streamline this process; these include systems that utilize pneumatic stepper motors powered by compressed air (in order to remain MRI compatible) to adjust a needle guide from within the control room, thereby removing the need for the patient to be removed from the scanner and the needle guide manually adjusted between each set of images [31,32].

The FUTURE trial compared cognitive, fusion, and in-bore MRI biopsy techniques in men with prior negative systematic biopsies and an ongoing suspicion of prostate cancer and found no difference in detection rates, though it was underpowered [33]. Other evidence is differing with no clear consensus on the best modality. Therefore, in-bore MRI biopsies do not currently seem to sufficiently justify the cost implications of the associated additional MRI time [34,35,36]. Though limited, initial evidence suggests that robot-assisted MRI-US fusion transperineal-targeted biopsies may have higher cancer detection rates and lower complications than cognitive-guided transperineal biopsies [28].

### 2.3. PET Imaging in Diagnosis

Molecular imaging in the form of positron emission tomography (PET) scanning has been used in the field of prostate cancer for some time. PET uses different radiolabelled tracers to identify and target specific biological pathways with a wide range and an increasing number of applications [37]. Radiolabels are positron-emitters; the emission of a positron leads to a positron-electron annihilation and subsequent production of two annihilation photons travelling in opposite directions. The annihilation photons can then be captured by the ring of detectors within the PET scanner. Conventional PET images have a resolution of around 4–5 mm and, as such, are usually performed in combination with a higher resolution modality to provide more detailed anatomical information, typically computed tomography (CT) [38].

Diagnostic accuracy in PET is dependent on the radiotracer used, with different tracers appropriate in different applications. In prostate cancer, several radiotracers have been trialled, including Choline, Fluciclovine, and prostate-specific membrane antigen (PSMA) [37]. Choline PET/CT, for example, has been studied in the primary diagnosis of prostate cancer, though it was seen to produce high rates of false negative and false positive results due to poor uptake in some tumours and excessive uptake in benign prostate tissue, with sensitivity and specificity in one study found to be 66% and 81%, respectively [39,40]. However, PSMA is a membrane-bound glycoprotein expressed predominately on prostate epithelial cells and shows increased expression in prostate cancer [41,42].

Whilst PSMA is primarily found within the prostate gland, its expression elsewhere has become increasingly recognized with the potential for false positives. It can be found in the vascular endothelium (and to a lesser extent, the tumour cells) of a number of other primary malignancies, which include other adenocarcinomas (breast, colorectal, pancreatic, and gastric), renal cell carcinoma, non-small cell lung cancer, glioblastoma multiforme, and transitional cell carcinoma [43]. Its presence can also be found in a range of normal tissue, such as the salivary glands, kidneys, bowel, spleen, and liver. Additionally, benign conditions, such as sarcoidosis or Paget’s disease and benign lesions, including meningiomas or haemangiomas, have been shown to cause false positive results [44,45]. In contrast, PSMA is not expressed in the same way in neuroendocrine prostate cancer, an aggressive variant of the disease, which can lead to false negatives [44].

Another consideration is the effect of androgen deprivation therapy (ADT) on PSMA expression. The effect of ADT appears to be dependent on the type of disease and scan timing, though there are some mixed results. Studies have shown a positive association between ADT use and tumour detection in the setting of recurrent disease [46]. In patients with castrate resistance metastatic disease, an increase in PSMA uptake following ADT commencement has been reported in multiple studies as variable but more pronounced within bony metastases [47,48]. Other studies looking at the treatment of hormone naïve patients with PSMA imaging at longer intervals of around 3 months post ADT have shown a reduction in tracer uptake, presumably corresponding with treatment effect [49,50].

PSMA is commonly targeted using PSMA-11 ligand in combination with the radionucleotide gallium-68 (Ga^68^-PSMA-11), which has a half-life of 67.7 min [37]. Along with the ^11^C-Choline and ^18^F-Fluciclovine radiotracers, it has an established role in the re-staging of patients with a biochemical relapse as part of a PET/CT. However, more recently, PSMA has been investigated in combination with mpMRI as a means of diagnostic imaging. The use of mpMRI over CT has the advantage of the improved anatomical differentiation and the ability to correlate radiotracer uptake with functional MRI sequences, such as DWI. The evidence comparing imaging results and pathology has shown that PSMA PET/MRI has superior diagnostic performance and tumour localization over mpMRI or PSMA PET alone, suggesting that its use as an additional diagnostic parameter is justified [51,52,53,54,55,56]. When used to target prostatic biopsies, a recent systematic review has shown that PSMA-PET (in combination with either CT or MRI) had a comparable diagnostic accuracy to mpMRI-targeted biopsies with a trend toward increased accuracy when mpMRI and PSMA-PET was used in combination, though it was limited by the lack of available evidence [57].

PRIMARY, a prospective Phase II trial, enrolled 291 biopsy naïve men with suspected prostate cancer. Participants underwent pelvic PSMA PET/CT and mpMRI, followed by systematic and targeted TP biopsy. It found that PSMA PET/CT combined with mpMRI had a higher negative predictive value and sensitivity than mpMRI alone, 91% vs. 72% and 97% vs. 83%, respectively [58]. A follow-up on the Phase III trial, PRIMARY2, is currently recruiting and aims to look at the men with a negative or equivocal MRI. In PRIMARY2, men will be randomized to either pelvic PSMA PET/CT with targeted biopsies if positive, or no biopsies if negative. This will be compared to the current standard of care of no additional imaging and template biopsy [59].

In renal cell carcinoma, it is commonplace for suspicious lesions on imaging to be treated with radical surgery without pathological confirmation. This is in part due to the high level of diagnostic accuracy of CT and a less acceptable non-diagnostic rate/negative predictive value associated with biopsy [60]. With increasing accuracy in diagnostic prostate imaging, it seems we may be nearing an era where proceeding directly to prostatectomy could be considered in select patients.

## 3. Role of Imaging in Active Surveillance

Low/intermediate-risk prostate cancer can be managed with active surveillance (AS) [6,61]. AS is a monitoring strategy whereby patients undergo a combination of regular physical examination, biochemical monitoring, and, when indicated, repeated mpMRI imaging and/or biopsies, with a view to definitive treatment should the disease progress. This allows patients to avoid or defer the associated morbidity definitive treatment brings, maintaining their quality of life, and it is associated with excellent long-term cancer-specific survival rates [62,63]. Active surveillance follow-up protocols vary. In the UK, the National Institute for Clinical Excellence (NICE) recommend PSA to be checked every 3–4 months in the first year and six monthly thereafter, with annual digital rectal examinations and a repeat MRI at 12–18 months with further MRIs and/or repeated biopsies indicated by concerning examination features or PSA kinetics [61].

mpMRI is used in active surveillance to assess for the progression of the disease in comparison with the baseline diagnostic MRI. This can be assessed formally using the Prostate Cancer Radiological Estimation of Change in Sequential Evaluation (PRECISE) criteria [64]. The PRECISE criteria aims to score the likelihood of disease progression on a scale of 1–5, where 1 or 2 represent disease regression, 3 is a stable disease, and 4 or 5 are varying degrees of radiological progression, with evidence showing that it performs well with a high specificity and positive predictive value (PPV) [64,65].

Previously, AS relied heavily on clinical examination and serial PSA to determine the need for re-biopsy. The relatively recent introduction of mpMRI potentially offers a less invasive alternative if it allows for biopsies to be omitted. However, two recent systematic reviews suggest that omitting biopsies and relying on mpMRI alone has insufficient diagnostic accuracy to exclude disease progression even when studies utilized the PRECISE criteria with a sensitivity and NPV for disease progression of 59–61% and 81–88%, respectively. Although, in one review, the use of the PRECISE criteria showed a non-significant trend toward improved performance [66,67]. That being said, when a reassuring mpMRI is combined with other contextualizing factors such as stable PSA kinetics and, in particular, a low PSA density (<0.15 ng/mL/cm^3^), there is evidence to suggest it may allow for repeated biopsies to be safely omitted [68].

Emerging methods to safely reduce repeated biopsies during active surveillance include the use of PSMA PET/CT. Current data on this application is limited, but one study has shown potential to reduce false positives and improve negative predictive value (NPV) compared with mpMRI [69].

## 4. Radiological Staging in Prostate Cancer

Prostate cancer is staged using the Tumour, Node, Metastasis (TNM) system, which assesses disease across each of its three criteria. It is used alongside PSA levels and histological grade to risk stratify disease and determine appropriate treatments.

Tumour (T) is assessed based on DRE findings, where T1 is impalpable, T2 is confined within the prostate, T3 extends beyond the prostate capsule, and T4 is an invasion into adjacent structures [6]. Whilst the T stage assessment is primarily clinical, it can also be assessed using mpMRI, which has been shown to be highly specific but only moderately sensitive for extraprostatic extension (T3a) and seminal vesicle invasion (T3b) [70,71]. Though MRI has limited sensitivity for picking up these adverse features, where found they have useful implications in their association with an increased risk of biochemical recurrence post-radical prostatectomy [72,73,74]. Traditionally, the risk of recurrence following definitive treatment was calculated using tools such as Partin’s tables. These allow for a risk assessment based on PSA, the clinical stage, and the Gleason score, and the evidence suggests the addition of the MRI into this assessment increases its accuracy [73,75]. An accurate assessment of risk preoperatively is important as it can lead to changes in surgical approach, such as the appropriate avoidance of nerve-sparing techniques to maximize oncological outcomes. However, whether the routine use of pre-operative staging mpMRI improves oncological outcomes in practice is unclear with conflicting evidence at present [76,77,78].

Nodal staging is assessed based on whether regional lymph node (LN) metastasis is present (N1) or not (N0), with non-regional lymph nodes upstaging to M1a [6]. Metastases are classified by the absence (M0) or presence (M1) of distant metastases, further stratified based on location with M1a assigned for non-regional lymph nodes, M1b for bony metastases, and M1c for metastases for other sites.

Patients at high risk of advanced disease are traditionally assessed with a combination of contrast CT of the abdomen and pelvis, primarily to identify nodal metastasis and technetium 99 (Tc^99^) bone scan, which examines for bone metastases by way of the increased radiotracer uptake in areas of high bone turnover. However, an accurate assessment of regional lymph nodes using conventional imaging is poor, with both CT and MRI having a low sensitivity of LN metastasis of 42% and 39%, respectively [79]. Additionally, whilst Tc^99^ bone scans are sensitive for bone turnover, this can lead to false positives and has the potential to miss early metastasis [80]. Although specificity can be increased with the addition of Single Photo Emission Computed Tomography (SPECT), alternative methods, such as whole-body MRI and PET/CT, have been shown to be superior in the detection of bony metastasis [81,82,83]. In a systematic review by Zhou et al., PSMA-PET/CT was found to have the highest sensitivity and specificity (97% & 100%) compared with whole-body MRIs (91% & 96%) or bone scintigraphy (86% & 95%) for detecting bone metastases on a per-patient basis [84]. More recently, a large RCT, proPSMA compared conventional imaging (CT and bone scan) with Ga^68^-PSMA-11 PET/CT and found the latter to have a higher sensitivity (85% vs. 38%) and specificity (98% vs. 91%) for the detection of a pelvic nodal or distant metastases [85]. Furthermore, a more recent Phase 2/3 trial OSPREY evaluated the diagnostic accuracy of 18F-DCFPyL-PSMA positron emission tomography/computerized tomography for pelvic lymph node involvement in 252 men undergoing a radical prostatectomy with extended pelvic lymph node dissection. They reported a sensitivity and specificity of 40% and 98%, respectively, though this improved to 60% and 97.9% for nodes >5 mm [86].

Accurate nodal staging is important as patients with N1M0 disease have a high risk of recurrence and, therefore, should be considered for adjuvant therapies dependent on nodal volume [6,87]. Despite the advances in imaging described above, extended pelvic lymph node dissection (ePLND) during radical prostatectomy remains the gold standard of local nodal staging, though this comes at the cost of a higher morbidity compared with a limited dissection [88]. ePLND and PSMA PET/CT have been directly compared with the former found to be significantly more sensitive [89]. Whilst it may not obviate the need for ePLND, predictive models using MRI, along with other factors, have been used to help select out which patients require ePLND [90]. Similar studies using PSMA PET/CT have shown that, combined with low-risk features, it can help avoid ePLND. However, a negative PSMA PET/CT in the high-risk group still necessitates the procedure [91].

Potential novel applications of PSMA imaging include radioguided surgery utilizing preoperative PSMA PET/MRI and intraoperative gamma probe to target avid lesions directly during robot-assisted radical prostatectomy [92]. Whilst extended lymph node dissections have been shown to have little oncological benefit, this technology presents a way to potentially increase accuracy whilst minimizing morbidity [88,92].

PSMA PET/CT’s routine use in the staging pathway is likely to increase the cohort of patients who have pelvic nodal and/or low volume metastasis (often termed oligometastatic disease), owing to its increased sensitivity in these patients. The treatment pathway for these patients is unclear, particularly regarding the role of localized treatment i.e., prostate radiotherapy or radical prostatectomy—often termed cytoreductive prostatectomy in this context. Current available evidence includes subgroup analyses from arms of the STAMPEDE trial, which examined the benefit of local prostate radiotherapy for patients with metastatic prostate cancer. Whilst no difference in overall survival was observed with the addition of radiotherapy, in a subgroup analyses looking only at patients with low-volume disease (low metastatic burden defined as less than four bones and no visceral metastases) a significant survival benefit was observed: 81% vs. 73% at 3 years [93]. There are a number of smaller retrospective studies examining the role of cytoreductive prostatectomy or radical radiotherapy in metastatic prostate cancer, and a recent systematic review examining these found that cytoreductive prostatectomy was associated with a significantly higher overall survival than systematic therapy (OR 2.54 at 5 years) and which was comparable to radiation therapy [94]. The other evidence includes a large population study using Surveillance Epidemiology and End Results (SEER) data from 2004–2010, examining the role of brachytherapy or radical prostatectomy in metastatic disease, which showed an increased survival in patients undergoing these treatments compared with those receiving no surgery or radiotherapy [95].

Further high-quality evidence is required to help further define the role of PSMA PET/CT in staging and the appropriate treatment for the low-volume metastatic prostate cancer cohort. The TROMBONE prospective randomized feasibility trial assessed radical prostatectomy for patients with oligometastatic disease and found that an RCT in this context is feasible. A number of trials are being conducted in this area at present. IP2-ATLANTA is one such RCT in progress, comparing the standard of care to a minimally invasive ablative therapy, cytoreductive prostatectomy, or radiotherapy [96,97].

## 5. Detection of Recurrent Disease

Recurrence following definitive treatment is typically monitored using PSA. In instances where recurrence has been noted, biochemically treatment options vary based on the original treatment received and whether the recurrence is visible radiologically. The latter has become increasingly important, with early evidence suggesting a survival benefit in those patients with oligometastatic disease who receive metastasis-directed therapy (MDT), which can include surgery or stereotactic body radiotherapy (SBRT) [98,99]. Early detection is also important with higher rates of curative salvage therapy seen when treatment is undertaken at low PSA levels.

This setting is perhaps the best established for PET/CT imaging. Studies have shown PSMA PET/CT to have a high level of accuracy, as it is able to detect recurrence at lower PSA levels than conventional imaging [86,100,101]. Among these, CONDOR, a Phase III trial, enrolled over 200 men with suspected recurrent prostate cancer and negative or equivocal conventional imaging. It found that PSMA-PET was able to detect a lesion in over 60% of these patients and that almost two-thirds of these patients underwent a change in management as a result [101]. Likewise, the OSPREY trial reported additional presumed metastatic disease in around 58% of patients with 18F-DCFPyL-PSMA PET/CT that conventional imaging was unable to detect in the recurrent setting. Additionally, they reported a sensitivity and specificity of 96% and 82%, respectively, for recurrent disease with 18F-DCFPyL-PSMA PET/CT [86]. There have been a number of recent systematic reviews performed to assess diagnostic performances of PET/CT in the recurrent disease setting. Wang et al. compared detection rates of ^18^F labelled fluciclovine, choline, and PSMA radiotracers and found PSMA to be better than fluciclovine and choline. This was most pronounced at low PSA levels with detection rates of 58% for PSMA vs. 35% and 23% for choline and fluciclovine, respectively [102]. Other systematic reviews have similarly concluded that PSMA is the superior choice of radiotracer. However, there are several different radiolabels that can be utilized [103,104,105,106,107].

The two most commonly used radiolabels for PSMA are gallium-68 [^68^Ga] or fluoride-18 [^18^F] [108,109]. A recent systematic review by Evangelista et al. found limited head-to-head evidence between the two radiolabels but noted a number of factors to take into account. For example, whilst ^68^Ga has the largest evidence base, it has a short half-life of 68 minutes, which can make distribution difficult unless on site or nearby ^68^Ga generators are available [110]. Furthermore, it has a high positron energy that may limit resolution, and it is primarily excreted via urine, which can limit the detection of small-volume disease adjacent to the urinary tract. In contrast, ^18^F has a half-life of 110 min, a low positron energy, and is primarily excreted by the liver, which can improve the detection of locoregional recurrence but may reduce sensitivity for the detection of visceral metastases [110]. Although limited by significant heterogeneity, Ma et al. found in their systematic review looking at detection rates for different radiotracers in recurrent prostate cancer that ^18^F labelled PSMA had a significantly higher detection rate than ^68^Ga [103].

The majority of studies focus on the use of PET combined with CT. However, with its role expanding in primary diagnosis, PET/MRI has also been described in the recurrent disease setting where, like PET/CT, it has been shown to have high detection rates and performs well at low levels of PSA [111,112]. However, at present, PET/MRI has not been shown to have superiority over PET/CT in this setting [111,112].

### Metastasis Directed Therapy (MDT)

A large proportion of patients who are diagnosed with recurrent prostate cancer after primary treatment do so with a small number of metastases. Whilst they would previously have been treated with surveillance and androgen deprivation therapy, MDT was developed in an effort to treat this cohort more effectively.

Two early randomized trials assessed MDT in men with recurrent prostate cancer with oligometastases. The STOMP trial randomized 62 men to MDT or surveillance. The majority of those receiving MDT underwent SBRT (*n* = 25), though a small portion underwent salvage lymph node dissection (*n* = 5), and one patient underwent a visceral metastectomy [98]. A similar study, ORIOLE, randomized 54 men to SBRT (*n* = 36) or surveillance (*n* = 18) [113]. Both trials allowed for up to three metastases and measured outcomes as disease progression, though STOMP defined this as time to ADT and ORIOLE as progression based on PSA increase, radiological progression, initiation of ADT, symptomatic progression, or death [98,113]. ORIOLE utilized PSMA PET/CT scans to detect recurrence, whereas a limitation of STOMP is that they used choline PET/CT, which, as previously discussed, is less sensitive at detecting recurrent disease [98,113]. Both studies showed benefit of MDT with pooled long-term outcomes reported showing progression free survival of 11.9 months for the MDT cohort compared with 5.9 for those undergoing surveillance [114].

Further studies examining the role of MDT are underway, and results are awaited to further define the treatment pathway in these patients. These include ADOPT, which a randomized Phase III trial comparing MDT with or without 6 months of concurrent ADT in men with recurrent oligometastatic prostate cancer [115]. In a similar cohort, PEACE V-STORM is a multicentre randomized trial comparing MDT + ADT with or without whole pelvic radiotherapy [116]. Another trial examining an alternative systemic therapy is POSTCARD, which aims to compare SBRT to SBRT in combination with Durvalumab, an immunotherapy that aims to enhance the immune response generated by radiotherapy [117].

## 6. Theranostics in Prostate Cancer

Theranostics is a relatively new term, the definition of which is varied throughout the literature, with some authors even questioning its use entirely [118]. Nonetheless, its use has become commonplace, and in this context we would define it broadly as a combination of diagnostic and therapeutic interventions, one example of which is PSMA-Targeted radioligand therapy. PET imaging uses radioisotopes that emit positrons and, by virtue of immediate annihilation via interaction with an electron, emits two gamma photons that travel through tissue and are detected by the PET scanner detectors. In contrast, potential therapeutic applications can utilize the absorption of radiation into localized tissue as a form of treatment, therefore requiring short penetration distances, such as those seen with beta particles. An example of this is PSMA-targeted radionuclide therapy with radioisotopes such as lutetium-177 (^177^Lu), among those commonly used.

Early trials utilizing PSMA-targeted therapies have shown promising results. The TheraP trial compared PSMA-targeted therapy using ^117^Lu-PSMA-617 to Cabazitaxel in men with metastatic castrate resistance prostate cancer (mCRPC) as a second-line treatment following docetaxel. It found an improved PSA response (66% vs. 37%) and reduced adverse effects in the ^117^Lu-PSMA-617 arm [119]. The VISION trial also studied PSMA-targeted therapy using ^117^Lu-PSMA-617 in men with mCRPC. Specifically, it looked at those who had ongoing disease progression despite treatment with both ADT and chemotherapy. Of note, patients who had only received one taxane therapy were ineligible if they were a candidate to receive a second. It compared patients receiving standard care alone (limited by the trial protocol to not include chemotherapy, radium-223, or immunotherapy), and those receiving PSMA-targeted therapy with ^117^Lu-PSMA-617 in addition to standard care. It found that PSMA-targeted therapy delayed progression and improved overall survival (15.3 vs. 11.3 months) when used in addition to standard care, and that it was safe and well-tolerated [120]. At present, based on the current evidence, the EAU consensus is that its use outside of clinical trials should be limited to patients with mCRPC [121]. Where exactly it fits within the pathway of treatment for these patients remains unclear at present. However, several trials are in progress, including those examining its use prior to taxane therapy, alongside a variety of other therapies and in metastatic hormone sensitive prostate cancer (mHSPC).

The recognized limitations of this approach include the reliance on PSMA expression to effectively target. This can be quantified on diagnostic PSMA PET/CT using standardized update values (SUV) with an increased response to treatment seen in those with a higher SUV and no response in low levels [122]. As a result, PSMA lesion positivity has been used as inclusion criteria for some clinical trials, though the definition used has varied. The TheraP trial restricted inclusion to PSMA positivity with an SUVmax (the maximum standardised uptake value) of at least 20 at a site of disease and SUVmax > 10 at all other measurable metastatic disease [119]. The VISION trial required at least one PSMA-positive metastatic lesion where PSMA positivity was defined as an uptake greater than that within the liver [120]. Additionally steps were taken to exclude PSMA negative disease; TheraP patients also underwent ^18^F-FDG PET/CT, and those with FDG positive and PSMA negative disease were excluded, whereas VISION excluded patients with PSMA uptake equal/lower than within liver in metastasis above a predefined size (>2.5 cm in lymph nodes or >1 cm in solid organs or bone lesions) [119,120]. Within VISION, 95.1% had a positive lesion, with 8.7% of patients excluded for PSMA negative lesions [120]. In TheraP, 10% of those screened were excluded for not meeting PSMA uptake criteria, and a further 18% due to discordant FDG uptake [119]. Though a standardized criteria does not exist for eligibility for PSMA targeted therapy, these are important considerations with prognostic implications, as patients who exhibit low PSMA uptake or FDG discordant lesions have been shown to have poor outcomes [123].

Alternative PSMA-targeting therapies being developed include immunotherapy. One example of this is pasotuxizumab, which binds to PSMA and to T cells resulting in the T-cell-mediated destruction of PSMA expressing cells [124]. This, and other similar therapies, are still early in their stages of development [124,125].

## 7. Conclusions

The field of prostate cancer imaging is an exciting one. There is ongoing development in areas of existing well-established applications, such as diagnosis and biopsy targeting. Perhaps more exciting still is the new areas of development, in particular, the future role of theranostics in the treatment pathway. The types of imaging modality and their role in diagnosis and treatment of prostate cancer discussed within this review are summarized in Table 2.

## Figures and Tables

**Table 1:** Summary of key trials examining mpMRI is prostate cancer diagnosis/biopsy.

							Clinically Significant Cancer			Clinically Insignificant Cancer	
	Year	Design		Cohort Size		Clinically Significant Definition	MRI ^a^ Sensitivity	TRUS ^b^ Biopsy Sensitivity			
PROMIS [5]	2017	Biopsy Naive	All men, MRI + TRUS + TP ^c^ Grid	576		Gleason score ≥ 4 + 3 or cancer containing core ≥ 6 mm	93%	48%			
				**MRI TB ^d^**	**SB**		**MRI TB Detection Rates**	**SB ^e^ Detection Rate**	**Combined Detection Rate**	**MRI TB Detection Rates**	**SB detection rate**
PRECISION [24]	2018	Biopsy Naive	RCT ^f^	252	248		38%	26%		9%	22%
MRI-FIRST [25]	2019	Biopsy Naive	SB + TB (cognitive or US fusion)	275		Gleason score ≥ 3 + 4	32.3%	29.9%	37.5%		
PAIREDCAP [26]	2019	Biopsy Naive	SB + TB (Cognitive and US ^g^ Fusion)	248		Gleason score ≥ 3 + 4	62.1%	60.1%	70.2%	24%	23%
4M [27]	2019	Biopsy Naive	SB + TB (MRI in-bore)	626		Gleason score ≥ 3 + 4	25%	23%		28%	25%

^a^ Magnetic Resonance Imaging, ^b^ Transrectal Ultrasound ^c^ Transperineal ^d^ Targeted Biopsies, ^e^ Systematic biopsies, ^f^ Randomized controlled trial ^g^ Ultrasound.

**Table 2:** Summary of Imaging modalities and their role in Prostate Cancer.

Modality	Setting				
	Diagnosis	Active Surveillance	Staging	Detection of Recurrent Disease	Theranostics
CT ^a^	-	-	Current Standard	-	-
MRI ^b^	Current Standard (mpMRI ^c^)	Current Standard (mpMRI)	Current Standard (mp/whole body MRI)	-	-
Tc99 ^d^ Bone scan	-	-	Current Standard	-	-
SPECT ^e^	-	-	Current Standard	-	-
PSMA PET ^f^/CT	Evolving Role	Evolving Role	Evolving Role	Current Standard	Evolving role (Lu^177^ targeted therapy)
PSMA PET/MRI	Evolving Role	-	-	Evolving Role	-

Current standard indicated in Green. Evolving Role indicated in Yellow. ^a^ Computed Tomography, ^b^ Magnetic Resonance Imaging, ^c^ Multiparametric MRI, ^d^ Technetium 99, ^e^ Single Photo Emission computed tomography, ^f^ Prostate Specific Membrane Antigen Position Emission Tomography.
